# Large Complex Odontoma of Mandible in a Young Boy: A Rare and Unusual Case Report

**DOI:** 10.1155/2014/854986

**Published:** 2014-04-10

**Authors:** G. Siva Prasad Reddy, G. V. Reddy, B. Sidhartha, K. Sriharsha, John Koshy, Rehana Sultana

**Affiliations:** Department of Oral & Maxillofacial Surgery, Panineeya Institute of Dental Sciences, Road No. 5, Kamala Nagar, Dilsukhnagar, Hyderabad, Andhra Pradesh 500060, India

## Abstract

Odontomas are the most common odontogenic tumors. They are broadly classified in to Compound Odontoma and Complex Odontoma. Among them complex odontoma is a rare tumor. Occasionally this tumor becomes large, causing expansion of bone followed by facial asymmetry. Otherwise these tumors are asymptomatic and are generally diagnosed on radiographic examination. We report a rare case of complex odontoma of mandible in a young boy. The tumor was treated by surgical excision under general anesthesia.

## 1. Introduction

Odontoma is a benign odontogenic tumor. The term odontoma was first coined by Broca (1866); he defined it as a tumor formed by an overgrowth of complete dental tissues [1]. Based on gross, radiographic, and microscopic features, odontomas are classified into complex odontoma and compound odontoma. WHO defines complex odontoma as malformation in which all of the dental tissues are represented, and individual tissues mainly are well formed but occur in disorderly pattern [2].

Odontomas constitute 22% of all odontogenic tumors. They occur in the first and second decade of life [3]. 70% of odontomas are associated with pathologic changes such as impaction, malpositioning, aplasia, malformation, and devitalization of adjacent teeth. Compound odontoma is twice as common when compared to complex odontoma. 60% of complex odontomas occur in women [4]. Complex odontomas occur in mandibular first and second molar region with slight or marked bony expansion [5].

The treatment of choice is surgical excision of the lesion followed by histopathological study to confirm the diagnosis. We present an interesting case of large complex odontoma of mandible in a young boy with marked bony expansion and impaction of lower second molar.

## 2. Case Report

A 13-year-old male patient presented with a chief complaint of swelling in the right lower jaw region for 6 months. The patient had no history of trauma.

**Figure 1:**
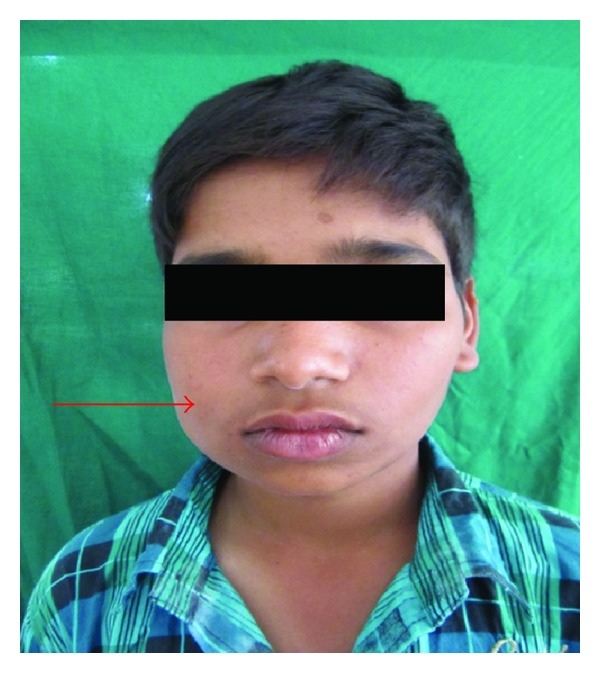
Preoperative frontal view showing swelling over right lower jaw.

Extraoral examination revealed a solitary swelling measuring 4 × 3 cm, present on the right lateral aspect of mandible extending from midway of the body to the ramus (Figure 1). On palpation the swelling was tender, hard in consistency, noncompressible, and nonreducible.

**Figure 2:**
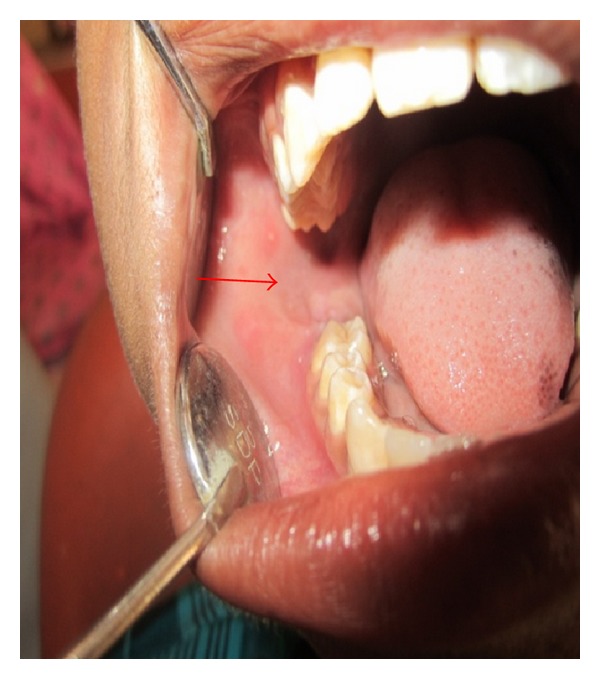
Intraoral photograph of lesion on right side of mandible.

Intraoral examination revealed missing mandibular right second molar tooth. There was slight discontinuity in mucosa distal to the right first molar (Figure 2). On palpation there was vestibular tenderness in relation to right mandibular first molar and palpable sharp point distal to the first molar. OPG and CT scans were advised.

**Figure 3:**
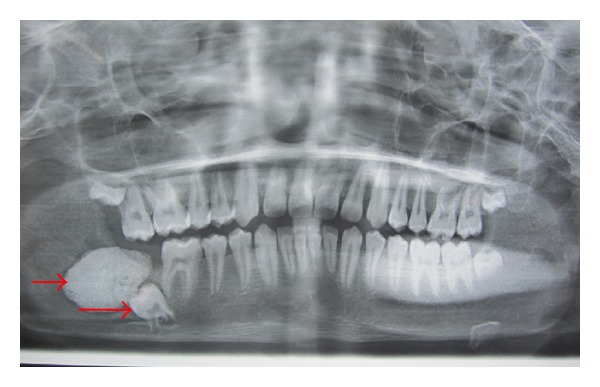
Orthopantomograph revealing radioopaque lesion in the right mandibular angle region involving the impacted second molar.

**Figure 4:**
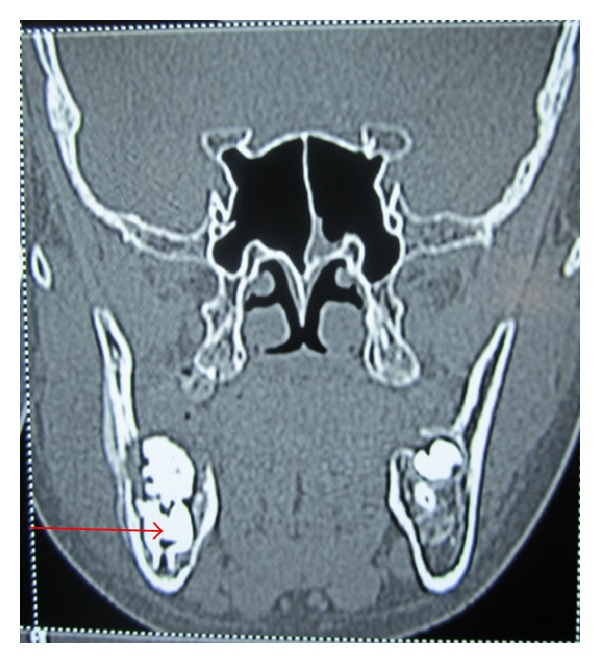
Coronal CT scan section showing the lesion.

**Figure 5:**
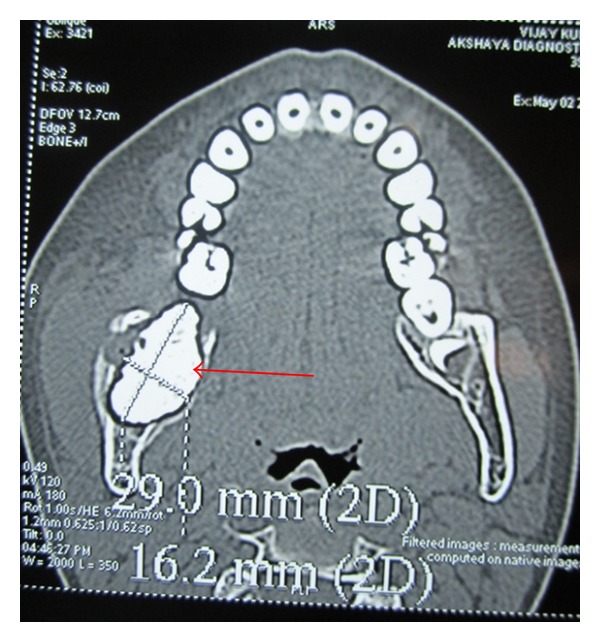
Axial CT scan section showing the extent of the lesion anteroposteriorly.

OPG revealed a radioopaque mass surrounded by a thin radiolucent line. The superior part of the mass protruding towards the superior border of the mandible. An impacted second molar with 1/4th root development was also seen (Figure 3). CT scan revealed both lingual and buccal cortical plate perforations and the extent of the lesion in anteroposterior direction (Figures 4 and 5). A provisional diagnosis of complex odontoma was made. Ameloblastic fibroodontoma and ameloblastic odontoma were considered in differential diagnosis.

**Figure 6:**
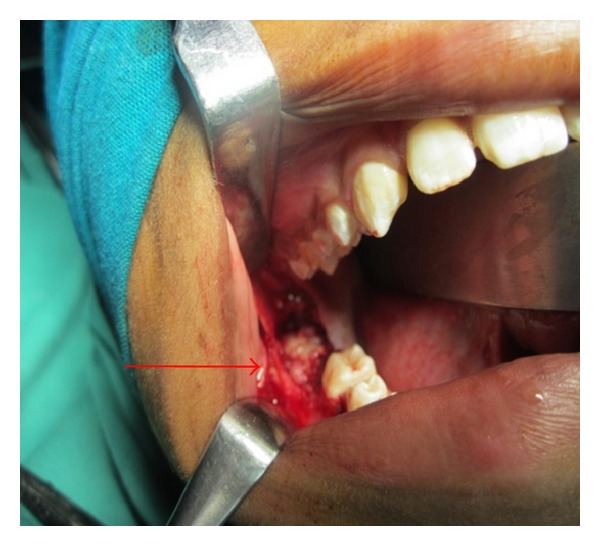
Intraoperative photograph showing the exposed lesion.

**Figure 7:**
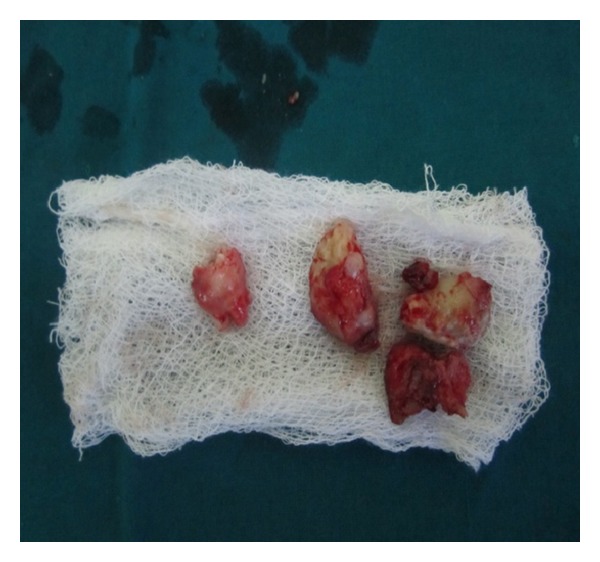
Excised specimen.

Under general anesthesia, the lesion was approached intraorally (Figure 6), and it was completely enucleated along with extraction of impacted second molar. After thorough curettage, the wound was closed using 3–0 vicryl and the specimen was sent for histopathological examination (Figure 7). Histopathological examination confirmed the diagnosis of complex odontoma. The patient was under observation for 6-month period. Postoperative OPG revealed good bony healing without any recurrence (Figure 9). Intraoral healing was good without any defects (Figure 10). There was no anaesthesia or paraesthesia of the areas supplied by inferior alveolar nerve on right side post operatively.

## 3. Discussion

Complex odontoma is a common odontogenic tumor, and it is usually a hard painless mass, which rarely exceeds diameter of the tooth. Most of these lesions are discovered accidentally on radiographic examination. The common signs and symptoms include impacted permanent teeth and swelling. Budnick found that 61% of cases are associated with impacted teeth [6].

The origin of complex odontoma is unknown; some suggest trauma or infection to be the cause. In a study conducted by Lopez-Areal et al., they found that a child developed multiple odontomas after experiencing trauma with intrusion of incisor teeth at the age of 10 months [7]. Hitchin has said that odontomas are inherited or developed as a result of genetic mutation [8]. An increased number of odontomas were found in people with Gardner's syndrome which is a heritable syndrome [9]. Recurrence of complex odontomas is very rare.

**Figure 8:**
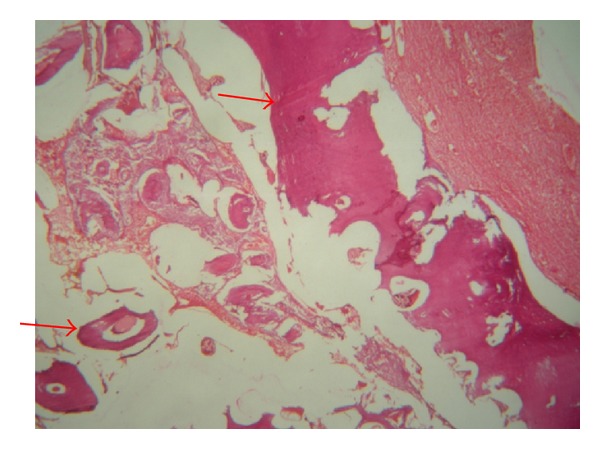
Photomicrograph showing scattered dentin in fibrous tissue. Periphery reveals continuous band of dentin, covered by layer of fibrous tissue.

**Figure 9:**
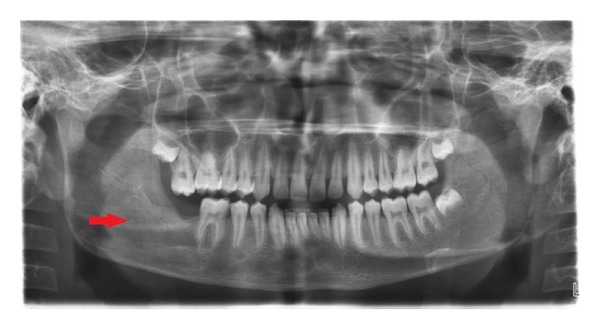
Six-month postoperative orthopantomograph revealed complete bony healing without any recurrence.

**Figure 10:**
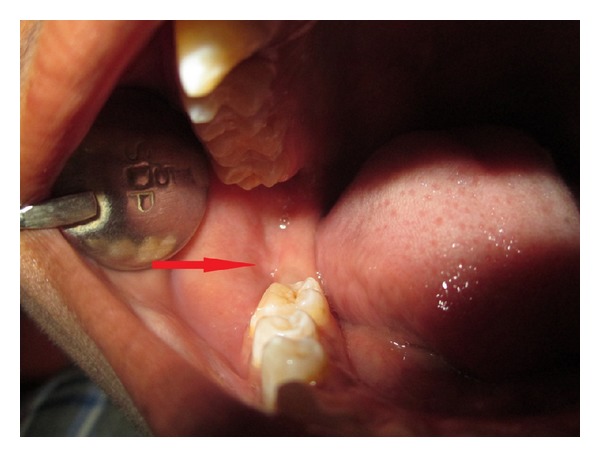
Postoperative intraoral photograph showing good healing of the surgical site.

Radiographically, complex odontoma appears as a radioopaque mass which does not resemble tooth structure. Histologically the complex odontoma is characterized by sheets of immature tubular dentin with encased hallow tooth like structures. Ghost cells are especially seen in complex odontoma (Figure 8). Conservative surgical excision of the lesion is the treatment of choice.

## 4. Conclusion

Odontomas in general are common, but complex odontomas are rare when compared to other odontomas. Complex odontomas should be surgically excised because they are characterized by expansion of cortical plates and if left untreated can cause pathological fracture of the bone.

## References

[1] Broca P (1866). *TraiteV Des Tumeurs*.

[2] Kramer IRH, Pindborg JJ, Shear M (1992). The WHO histological typing of odontogenic tumours. *Cancer*.

[3] Bhaskar SN (1981). *Synopsis of Oral Pathology*.

[4] Wood NK, Goaz PW (1985). *Differential Diagnosis of Oral Leasionsed*.

[5] Bodin I, Julin P, Thomsson M (1983). Odontomas and their pathological sequels. *Dentomaxillofacial Radiology*.

[6] Budnick SD (1976). Compound and complex odontomas. *Oral Surgery Oral Medicine and Oral Pathology*.

[7] Lopez-Areal L, Donat FS, Gil Lozano J (1992). Compound odontoma erupting in the mouth: 4-year follow-up of a clinical case. *Journal of Oral Pathology and Medicine*.

[8] Hitchin AD (1971). The aetiology of the calcified composite odontomes. *British dental journal*.

[9] Hisatomi M, Asaumi J-I, Konouchi H, Honda Y, Wakasa T, Kishi K (2002). A case of complex odontoma associated with an impacted lower deciduous second molar and analysis of the 107 odontomas. *Oral Diseases*.

